# The effect of facial muscle contractions on the cerebral state index in an ICU patient: A case report

**DOI:** 10.1186/1757-1626-1-167

**Published:** 2008-09-19

**Authors:** Shahram Borjian Boroojeny

**Affiliations:** 1 Anesthesiology department, medical school, Zahedan university of medical sciences, Zahedan, Iran

## Abstract

**Introduction:**

Cerebral state monitor is a monitor which shows depth of anesthesia in a number between 0–100 as cerebral state index, in which 40–60 is appropriate for general anesthesia. The effect of electromyogram on cerebral state index has not been shown yet.

**Case report:**

A 24-year-old Iranian-balooch man admitted in the intensive care unit because of head injury in a car accident. In spite of sustained low level of consciousness, his cerebral state index had significant fluctuations coordinated with electromyogram resulted from facial muscle contractions. After neuromuscular blocking agent prescription, cerebral state index was decreased from about 90 to 40, directly followed the changes in electromyogram.

## Background

The Cerebral State Monitor (Danmeter A/S, Odense, Denmark) is a portable, wireless monitor that uses the time and frequency domain analysis, which inputs into a fuzzy logic inference system to show a 0 to 100 scale, the Cerebral State Index (CSI), with 40 to 60 indicating an adequate depth of hypnosis [1]. In a study, the CSI had a predictive probability statistic for depth of anesthesia of 0.87, which demonstrates good performance [2]. Moreover the CSI performed better for deeper levels of anesthesia than the other brain monitor, BIS(Aspect Medical Systems, Norwood, MA, USA), which was better at lighter levels. There is not any report about the effect of the electromyogram (EMG) signal which may artificially increase the CSI number. EMG signal is high frequency, but it is possible to overlap with low frequency CSI signal. Such interference can be seen with another monitor of depth of anesthesia, BIS [3,4]. If the CSI is shown artificially high in an ICU patient, probably the sedation of the patient will be decreased which can lead to premature awakening and increase intra cerebral pressure and death. So understanding the limitations and disturbing factors of the CSI which is a useful monitor in the ICU and operating theater, is very important, because wrongly decision making on the basis of a false CSI can be life threatening.

## Case report

**Figure 1:**
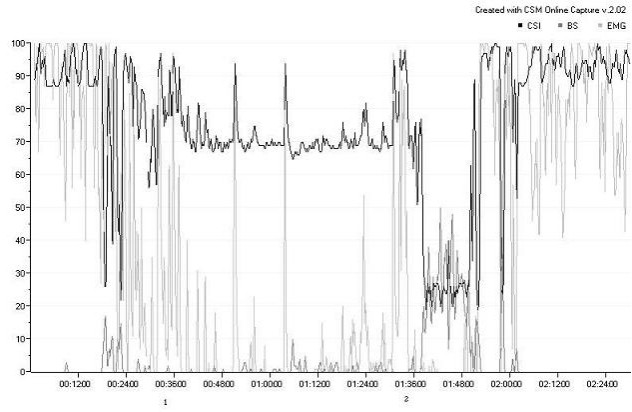
**CSI curve of the patient**. CSI: cerebral state index, BS: burst suppression, EMG: electromyogram. 1: first injection of atracurium (25 mg), 2: second injection of atracurium(15 mg). As appeared in the figure, CSI changes follow the EMG changes. BS increases when EMG decreases.

A 24-year-old Iranian-balooch man was admitted in the ICU because of head injury. He had not any history of medical or surgical diseases before the accident. His CT scan showed generalized brain edema, the Glasgow coma scale score (GCS) on arrival was 4. In the ICU the patient was sedated by remifentanil and midazolam to decrease the brain edema via hyperventilation and decreasing blood pressure. Twenty four hours later, the CSI was appeared with fluctuations that correlated to muscular twitching in the facial muscles. The CSI had also fluctuations parallel to EMG activities which appeared on the CSM screen with a short time delay, less than one minute (figure 1) . When EMG activity reached to 100%, CSI became 100, and when it went down to 25%, CSM also became 25–30. All of the CSI data was recorded automatically and wirelessly by the company's standard software. Because of the patient's brain condition; it was very important to evaluate the level of consciousness. So, atracurium 25 mg intravenously was injected at 00:36 a.m leading to disappearing EMG activity three minutes later except sporadic surges. Concomitant with decreasing EMG, CSI was decreased from 100 to 70. An interesting finding was increasing burst suppression (BS) from zero to about 10% after first injection of atracurium. After returning EMG at o1:34 a.m, the second dose of atracurium was injected 15 mg intravenously at 1:36 leading to complete EMG recession and increasing BS to 20–30% and decreasing CSI to 20–40. All of the time GCS was 7 and Richmond Agitation-Sedation Scale remained between 0 to 1.

## Discussion

The aim of the Cerebral State Index (CSI) is to monitor the level of consciousness or hypnosis during general anesthesia or in the ICU. The CSI is a unitless scale from 0 to 100, where 0 indicates a flat electroencephalographic signal and 100 indicates the awakening state. The 40–60 range is adequate range for anesthesia. The CSI requires three electrodes positioned at the middle forehead, left forehead, and left mastoid. Alternatively, the right forehead and right mastoid can be used.

The CSI is calculated based on four sub parameters of the electroencephalogram:

Alfa ratio, Beta ratio, Alfa ratio – Beta ratio, and burst suppression, calculating an index from 0 to 100. The novelty of the CSI is that a fuzzy inference system was used in Inference System (ANFIS). During burst suppression, the Alfa and Beta ratios are no longer monotonously decreasing as a function of anesthetic depth, and therefore, they cannot be used in the calculation of the final index [4]. Calculating CSI needs high frequency EEG as well as low frequency, the high frequency component of EEG (that is above 30 Hz) overlaps with EMG frequency starting from 30 Hz up to 42.5 Hz, which is the upper limit of EEG usage for CSI calculation [5]. To our knowledge, the misinterpretation of EMG as EEG can be occurred in Beta Ratio component of bispectral index(BIS), but there not any published papers about such effect regarding to CSI [5]. Omitting or filtering out the 30 Hz or more frequency component of EEG is not acceptable because of the importance of the component in evaluating of hypnosis. Using neuromuscular blocking agents in clinical and short period probably can not directly affect depth of hypnosis, and its monitoring, except response entropy, which is the only commercially available depth of anaesthesia index in which facial EMG activity is included in the calculation algorithm [5].

## Conclusion

In conclusion, CSI can be used for detecting depth of anesthesia or sedation, but overlapping EEG with EMG is an important and sometimes very hazardous pitfall. Wrong assessment of depth of anesthesia can lead to administrate so many anesthetic or hypnotic drugs with many life threatening adverse effects in operating theatre, or misinterpretation of the brain condition of the patient causes premature weaning of the sedation and ventilatory support of the patient.

## Abbreviations

CSI: cerebral state index; CSM: cerebral state monitor; BIS: bispectral indexl; EMG: electromyogram; BS: burst suppressionl; EEG: electroencephalogram.

## Competing interests

The author declares that they have no competing interests.

## Authors' contributions

The single author was involved in the management of the case and finalizing the article. The single author was involved in the process of editing, correcting, and finalizing the manuscript. The author has read and approved the final manuscript.

## Consent

Written informed consent was obtained from the patient's family for publication of this case report and accompanying images. A copy of the written consent is available for review by the Editor-in-Chief of this journal.
